# The prevalence of atypical scrapie in sheep from positive flocks is not higher than in the general sheep population in 11 European countries

**DOI:** 10.1186/1746-6148-6-9

**Published:** 2010-02-07

**Authors:** Alexandre Fediaevsky, Cristiana Maurella, Maria Nöremark, Francesco Ingravalle, Stefania Thorgeirsdottir, Leonor Orge, Renaud Poizat, Maria Hautaniemi, Barry Liam, Didier Calavas, Giuseppe Ru, Petter Hopp

**Affiliations:** 1 AFSSA-Lyon, Unité Epidémiologie, 31 Avenue Tony Garnier, 69364 Lyon, France; 2 INRA Clermont-Theix, Unité épidémiologie animale, F63122 Saint Genès Champanelle, France; 3 Italian Reference Centre for Animal TSEs-Istituto Zooprofilattico Sperimentale del Piemonte, Liguria e Valle d'Aosta, Italy; 4 Department of Disease Control and Epidemiology, SVA, National Veterinary Institute, 751 89 Uppsala, Sweden; 5 Institute for Experimental Pathology, University of Iceland, Keldur v/Vesturlandsveg, IS-112 Reykjavík, Iceland; 6 Sector de diagnóstico de EETs, Laboratório de Patologia - Unidade de Sanidade Animal, Laboratório Nacional de Investigação Veterinária, INRB, I.P., Estrada de Benfica 701, 1549-011 Lisboa, Portugal; 7 AFSCA, DG Politique de Contrôle, Bd du Jardin Botanique, 55, 1000 Bruxelles, Belgium; 8 Veterinary Virology, Finnish Food Safety Authority, 00790 Helsinki, Finland; 9 DAFF Administration Building, Backweston Campus, Stacumney Lane, Young's Cross, Celbridge, Co. Kildare, Ireland; 10 Section of epidemiology, National Veterinary Institute, PO Box 750 Sentrum, 0106 Oslo, Norway

## Abstract

**Background:**

During the last decade, active surveillance for transmissible spongiform encephalopathies in small ruminants has been intensive in Europe. In many countries this has led to the detection of cases of atypical scrapie which, unlike classical scrapie, might not be contagious. EU legislation requires, that following detection of a scrapie case, control measures including further testing take place in affected flocks, including the culling of genotype susceptible to classical scrapie. This might result in the detection of additional cases. The aim of this study was to investigate the occurrence of additional cases in flocks affected by atypical scrapie using surveillance data collected in Europe in order to ascertain whether atypical scrapie, is contagious.

**Results:**

Questionnaires were used to collect, at national level, the results of active surveillance and testing associated with flock outbreaks in 12 European countries. The mean prevalence of atypical scrapie was 5.5 (5.0-6.0) cases per ten thousand in abattoir surveillance and 8.1 (7.3-9.0) cases per ten thousand in fallen stock. By using meta-analysis, on 11 out of the 12 countries, we found that the probability of detecting additional cases of atypical scrapie in positive flocks was similar to the probability observed in animals slaughtered for human consumption (odds ratio, OR = 1.07, CI_95%_: 0.70-1.63) or among fallen stock (OR = 0.78, CI_95%_: 0.51-1.2). In contrast, when comparing the two scrapie types, the probability of detecting additional cases in classical scrapie positive flocks was significantly higher than the probability of detecting additional cases in atypical scrapie positive flocks (OR = 32.4, CI_95%_: 20.7-50.7).

**Conclusions:**

These results suggest that atypical scrapie is not contagious or has a very low transmissibility under natural conditions compared with classical scrapie. Furthermore this study stressed the importance of standardised data collection to make good use of the analyses undertaken by European countries in their efforts to control atypical and classical scrapie.

## Background

Scrapie is a fatal neurodegenerative disease affecting sheep and goats which belongs to the group of diseases called transmissible spongiform encephalopathies (TSE). In its classical form, it is a contagious disease with susceptibility influenced by punctual mutations on the prion gene (*prnp*) coding for the prion protein (PrP) [1]. In 1998, a new type of scrapie called scrapie Nor98 was detected [2] and in 2005 the European Food Safety Authority (EFSA) defined diagnostic criteria for classical scrapie (CS) and for atypical scrapie (AS), including Nor98, based on the results of Western blot pattern of the pathogenic prion protein (PrP^Res^) [3]. Since the diagnosis of AS poses some specific difficulties because of proteinase K susceptibility and variable distribution of PrP^Res ^[4], EFSA also evaluated the sensitivity of the different TSE rapid tests to detect AS on different biological material (table 1) [5,6].

**Table 1:** Groups of detection of atypical scrapie according to rapid tests and material usually analysed (according to EFSA 2005)

Group	Sample	Rapid test
1	Brainstem with or without cerebellum or cerebrum	Either Biorad Te-SeE/Biorad Platelia, or Biorad Te-SeE Sheep and Goat, or IDEXX HerdChek BSE-Scrapie Antigen Test Kit, EIA

2	Brainstem and either cerebellum or cerebrum	Either Prionics-Check Western Small Ruminant, or Enfer TSE Version 3, or Enfer TSE Kit version 2.0 or Beckman Coulter InPro CDI, or POURQUIER - LIA Scrapie

3	Brainstem and either cerebellum or cerebrum	Prionics Check LIA Small Ruminants
	Brainstem only	Neither Biorad Te-SeE/Biorad Platelia, nor Biorad Te-SeE Sheep and Goat, nor IDEXX HerdChek BSE-Scrapie Antigen Test Kit, EIA

As a contagious disease, CS is often clustered within flocks and regions. Infected animals usually die at the end of the clinical course of the disease when they are between two to four years of age. Animals carrying PrP genotypes with V_136_R_154_Q_171 _and/or A_136_R_154_Q_171 _alleles are considered most susceptible to the disease [1]. In contrast to CS, AS is usually detected in older animals (mean age of five to six years) [4] and PrP genotypes that include alleles A_136_H_154_Q_171 _and/or A_136_F_141_R_154_Q_171_, are more at risk [7]. Although the disease has been shown to be experimentally transmissible by intracerebral inoculation to mice [8] and sheep [9], transmission between animals under natural conditions has not yet been demonstrated. AS has been reported to have scattered geographical appearance [10,11] and usually only a single affected animal in a flock has been detected [4]. Nevertheless, the occurrence of more than one AS case in individual flocks has been reported [4,11-13]. No factors demonstrating horizontal transmission were found in case control studies in Norway [10] or France [14] or by network analysis of movement data in the UK [15]. Furthermore three cases have occurred in an experimental flock presumed free from scrapie and with no explanation for any possible source of contamination [16]. Due to the different features of AS compared to CS it has been suggested that AS could develop without exposure to an infectious agent [4].

Since 2002, intensive active surveillance for TSE in healthy slaughter sheep, *i.e*. sheep slaughtered for human consumption, and fallen stock *i.e*. sheep which have died or been killed but not slaughtered for human consumption, has been performed within the European Union (EU). It has previously been shown that the prevalence of AS in slaughtered animals and fallen stock is similar throughout Europe [12]. When positive cases have been detected through active surveillance legislation requires control measures and further testing of animals in the associated flock [17] - flocks controlled in this way will be designated as "positive flocks" for the purposes of this paper. This can and has lead to the detection of further cases in positive flocks, designated as "additional cases" in this paper. European legislation which has been amended since first inception and common to all member states has allowed for the employment of different disease control options/strategies in member states. Specific provisions for control of AS were first introduced in July 2007 [18]. It should be emphasised, in relation to animal health, that the suitability of AS control measures will depend on whether the disease is contagious or not.

The aim of this study was to investigate, at national level, if the occurrence of additional cases of AS could help in clarifying the potential contagious nature of the disease. Insights have been gleaned by assessing surveillance data from different European countries and comparing prevalences in different streams of surveillance. The prevalence of AS among animals tested in AS positive flocks was compared with 1) the prevalence of AS in the healthy slaughter surveillance stream, 2) the prevalence of AS in fallen stock surveillance stream, 3) the prevalence of AS in sheep tested in CS positive flocks and 4) the prevalence of CS in sheep tested in AS and CS positive flocks.

## Results

From the seventeen countries that had reported at least one case of atypical scrapie from 2002 to 2007, twelve countries answered the questionnaire, three countries declined participation and two countries did not answer. The following eleven countries were included in the analysis: Belgium, Denmark, Finland, France, Greece, Iceland, Italy, Netherlands, Norway, Portugal and Sweden. The data from Ireland was presented but could not be included in some analyses for various reasons: AS prevalence was not estimated because different groups of screening tests/analyses (see table 1) were used in different streams and different years and CS prevalence was not estimated because for the earliest datasets a precise figure for the number of animals tested from CS positive flocks was unavailable.

### Control strategies

The applied control strategies by country are shown in table 2. Three main categories were defined for each type of scrapie: i) stamping out: all animals from the positive flocks were destroyed and either all or a sample of the adults were tested, ii) selective culling: animals were genotyped and animals carrying certain genotypes (those mostly susceptible to CS) were destroyed and tested, and iii) intensified monitoring: the flocks were not culled, but adults leaving the flocks as healthy slaughter or fallen stock were tested. Italy applied an extended selective culling in flocks with AS, and this was reported separately. Within each of the three categories, differences existed between countries *i.e*. in some instances animals tested from suspect flocks and contact flocks were included in the reporting, the minimum age of the tested animals varied, the proportion of animals tested among animals destroyed differed and differing genotypes were selected during selective culling and subsequently tested. There were also changes over time within the countries. Stamping out was applied in five countries for CS and seven countries for AS, selective culling was applied in six countries for CS and eight countries for AS and intensified monitoring was applied in one country for CS and seven countries for AS (table 2).

**Table 2:** Grouped control strategies reported by countries

		Intensified monitoring	Selective culling*	Other selective culling**	Stamping out
AS	Countries^§^	FR, GR, DK, FI, NO, PT, SE	FR, BE, IE, IT, NL, DK, NO, PT	IT	IE, IT, IS, DK, FI, NO, SE
	Suspect flocks included	FR, GR, DK, FI, PT	FR, BE, IT, DK,	IT	IE, IT, DK, FI
	Contact flocks included	FI, PT	BE, PT		FI, IS
	Stream under surveillance^!^	HS and FS: all countries	Culled animals: all countries HS in all countries except NL and DK and BE in some years FS in all countries	HS, FS and culled animals	Culled animals in all countries except IE no other stream except HS and FS in FI and FS in DK
	Animals above age limit tested	All animals	All animals except in BE (FS): only some animals	All animals	All animals except in IS: only some animals
	Age limit for testing	18 months except in FR and in the first years in PT and in SE: 12 months	Culled animals: 12 months except in NO: 18 months and in BE (unspecified) other stream 18 months except in FR, IT, NL and in the first years in PT: 12	18 months	12 months except in DK, FI and NO: 18 months
CS	Countries^§^	FR	FR, BE, GR, IE, IT, NL		GR, IE, IT, IS, NO
	Suspect flocks included	FR	FR, BE, GR, IT		GR, IE, IT
	Contact flocks included		BE		IS
	Stream under surveillance^!^	HS and FS	Culled animals in all countries HS and FS in all countries except HS in NL and in BE in some years		Culled animals in all countries no other stream except HS and FS in GR
	Animals above age limit tested	all animals	Culled animals: all countries except in BE, IT, NL: only some animals other stream: all countries except in BE: only some animals		All animals except in IE, IT, IS: only some animals
	Age limit for testing	12 months	Culled animals: 12 months except in BE (unspecified) Other stream: 18 months except in FR, IT and NL(FS): 12 months		12 months except in NO: 18 months

^§ ^BE: Belgium, DK: Denmark, FI: Finland, FR: France, GR: Greece, IE: Ireland, IS: Iceland, IT: Itlay, NL: Netherlands, NO: Norway, PT: Protugal, SE: Sweden, ^!^: HS: Healthy slaughter, FS: Fallen stock * all males not ARR/ARR and all females carrying a VRQ allele or not carrying an ARR allele, except in Italy where animals with AHQ and AFRQ were also culled and in Portugal where animals with ARR/AHQ genotype were culled but not those with ARH/ARH, ARH/ALRQ or ALRQ/ALRQ, ** all males carrying AHQ or AFRQ alleles, *** all males not ARR/ARR and all females carrying a VRQ allele or not carrying an ARR allele

### AS in active surveillance

Out of eleven countries that used combinations of rapid tests and samples recommended to detect AS (group 1 analyses in table 1) for active surveillance, ten countries had detected at least one positive case of AS in active surveillance of healthy slaughter while no positive case had been detected in The Netherlands. The prevalences, with a mean value of 5.5 (5.0 - 6.0) cases per ten thousand, are shown in table 3. In fallen stock, eight countries had detected at least one positive case of AS, with no cases found in Greece, Iceland and The Netherlands. The prevalences, with a mean value of 8.1 (7.3 - 9.0) cases per ten thousand are shown in table 3. The prevalences were significantly higher in fallen stock compared to healthy slaughter (results of the GLMM: odds ratio (OR) = 1.57, 95% confidence interval, (CI_95%_): 1.36 - 1.82). In The Netherlands, 1 AS case was detected out of 43,346 group 3 tests in healthy slaughter and 3 cases of AS were detected out of 42,622 group 3 tests in fallen stock. No other countries detected AS case in active surveillance with group 3 tests. In Ireland, 3 AS cases were detected but these cases were detected following clinical surveillance and associated testing regimes (histopathology and immunohistochemistry) rather than rapid screening testing (group screening tests).

**Table 3:** Prevalence of atypical scrapie (AS) in active surveillance from starting year for detection of AS in active surveillance to 2007

Surveillance stream	Starting year	Country	Cases	Tests	Prevalence (per 10,000)	CI 95%
Fallen stock	2004	Belgium	4	3238	12.4	3.4 - 31.6
	2006	Denmark	3	8177	3.7	0.8 - 10.7
	2004	Finland	4	3924	10.2	2.8 - 26.1
	2002	France	225	304832	7.4	6.4 - 8.4
	2006	Greece	0	7913	0	0 - 4.7
	2005	Iceland	0	152	0	0 - 239.8
	2005	Italy	11	27945	3.9	2.0 - 7.0
	2004	The Netherlands	0	1000	0	0 - 36.8
	2002	Norway	27	21137	12.8	08.4 - 18.6
	2004	Portugal	77	48662	15.8	12.5 - 19.8
	2004	Sweden	7	12434	5.6	2.3 - 11.6
						
Healthy slaughter	2004	Belgium	2	16109	1.2	0.2 - 4.5
	2006	Denmark	1	2476	4.0	0.1 - 22.5
	2004	Finland	1	5618	1.8	<0.1 - 9.9
	2002	France	190	318333	6.0	5.2 - 6.9
	2006	Greece	3	20467	1.5	0.3 - 4.3
	2005	Iceland	0	10931	0	0 - 3.4
	2005	Italy	32	86745	3.7	2.5 - 5.2
	2004	The Netherlands	0	2719	0	0 - 13.6
	2002	Norway	24	87753	2.7	1.8 - 4.1
	2004	Portugal	168	213923	7.9	6.7 - 9.1
	2004	Sweden	5	12359	4.0	1.3 - 9.4

### AS and CS in positive flocks

All countries had detected at least one case of AS and had subsequently tested sheep from AS positive flocks. Four countries (France, Iceland, Italy and Portugal) had detected additional cases with group 1 tests using the same diagnostic procedure in positive flocks and active surveillance. The mean national additional AS case prevalence in positive flocks was 6.7 cases per ten thousand. No significant difference existed between prevalences for different control strategies (selective culling versus intensified monitoring, results of the GLMM: OR = 0.7, CI_95%_: 0.33 - 1.84; stamping out versus intensified monitoring, results of the GLMM: OR = 1.58, CI_95%_: 0.36 - 6.94) (table 4).

**Table 4:** Prevalences of additional atypical scrapie (AS) cases by control strategy and country from flocks in which the first scrapie animal had either AS or classical scrapie (CS)

Strategy	Country	Cases	Tests	Prevalence (per 10,000)	CI 95%
Intensified monitoring in AS positive flocks	Finland	0	36	0	0 - 973.9
	France	11	14965	7.4	3.7 - 13.1
	Greece	0	67	0	0 - 535.7
	Norway	0	75	0	0 - 480.0
	Portugal	3	4612	6.5	1.3 - 19.0
	Sweden	0	47	0	0.0 - 754.9
Intensified monitoring in CS positive flocks	France	7	31113	2.2	0.9 - 4.6
Selective culling in AS positive flocks	Belgium	0	223	0	0 - 164.1
	Denmark	0	168	0	0 - 217.2
	France	5	10462	4.8	1.6 - 11.1
	Italy	1	2260	4.4	0.1 - 24.6
	The Netherlands	0	60	0	0 - 596.3
	Norway	0	1033	0	0 - 35.6
	Portugal	2	1763	11.3	1.4 - 40.9
Selective culling in CS positive flocks	Belgium	0	295	0	0 - 124.3
	France	4	12087	3.3	0.9 - 8.5
	Greece	3	10310	2.9	0.6 - 8.5
	Italy	2	8565	2.3	0.3 - 8.4
Special genetic selection in AS positive flocks	Italy	0	351	0	0 - 104.5
Stamping out in AS positive flocks	Denmark	0	17	0	0 - 1950.6
	Finland	0	173	0	0 - 211.0
	Iceland	1	467	21.4	0.5 - 118.7
	Italy	1	1309	7.6	0.2 - 42.5
	Norway	0	1156	0	0 - 31.9
	Sweden	0	195	0	0 - 187.4
Stamping out in CS positive flocks	Iceland	0	367	0	0 - 100.0
	Italy	0	3231	0	0 - 11.4
	Norway	0	343	0	0 - 107.0

CS cases were also detected in flocks with an index case of AS in France and Italy (table 5), with a mean of 5.3 cases per ten thousand. In Ireland, one additional case was detected out of 19 animals tested during stamping out measures in AS positive flocks and no additional cases were detected from 66 animals tested during further testing in AS positive flocks [13].

**Table 5:** Prevalence of additional classical scrapie (CS) cases by control strategy and country from flocks in which the first scrapie animal had either atypical scrapie (AS) or CS.

Strategy	Country	Cases	Tests	Prevalence (per 10,000)	CI 95%
Intensified monitoring in AS positive flocks	Finland	0	36	0	0 - 973.9
	France	0	20720	0	0 - 1.8
	Greece	0	67	0	0 - 535.7
	Norway	0	75	0	0 - 480.0
	Portugal	0	4612	0.	0 - 08.0
	Sweden	0	47	0	0 - 754.9

Intensified monitoring in CS positive flocks	France	203	51760	39.2	34.0 - 45.0

Selective culling in AS positive flocks*	Belgium	0	223	0	0 - 164.1
	Denmark	0	168	0	0 - 217.2
	France	3	18061	1.7	0.3 - 4.9
	Ireland	0	66	0	0 - 543.6
	Italy	6	2260	26.5	9.7 - 57.7
	The Netherlands	0	120	0	0 - 302.7
	Norway	0	1033	0	0 - 35.6
	Portugal	0	1763	0	0 - 20.9

Selective culling in CS positive flocks*	Belgium	7	295	237.3	95.9 - 482.8
	France	658	21438	306.9	284.2 - 330.9
	Greece	1054	10310	1022.3	964.5 - 1082.4
	Ireland	230	11441	201.0	176.1 - 228.4
	Italy	728	11750	619.6	576.6 - 664.7
	The Netherlands	181	4003	452.2	389.9 - 521.2

Special genetic selection in AS positive flocks	Italy	8	352	227.3	98.6 - 442.9

Stamping out in AS positive flocks	Denmark	0	17	0	0 - 1950.6
	Finland	0	173	0	0 - 211.0
	Iceland	0	467	0	0 - 78.7
	Ireland	0	19	0	0 - 1764.7
	Italy	1	1309	7.6	0.2 - 42.5
	Norway	0	1156	0	0 - 31.9
	Sweden	0	195	0	0 - 187.4

Stamping out in CS positive flocks	Iceland	81	3290	246.2	196.0 - 305.1
	Ireland	31	42768	7.2	4.9 - 10.3
	Italy	184	7415	248.1	213.9 - 286.2
	Norway	8	343	233.2	101.2 - 454.4

*Genotypes mostly susceptible to CS, see table 1

Eight countries also applied control strategies in CS positive flocks during the study period (Belgium, France, Greece, Iceland, Ireland, Italy, The Netherlands and Norway). All these countries detected CS additional cases (table 5) and the mean prevalence for CS additional cases the seven countries included in the analysis (Ireland excluded) was 280.6 cases per ten thousand. Selective culling prevalences were significantly higher compared to stamping out prevalences (results of the GLMM: OR = 2.27, CI_95%_: 2.19 - 3.02). Selective culling prevalences were also higher when compared to intensive monitoring (applied only in France, OR = 8.08, CI_95%_: 6.90 - 9.46). AS cases were also detected in flocks with a CS index case in France, Greece and Italy (table 4), with a mean of 2.4 cases per ten thousand. The prevalence rates were not significantly different between the different control strategies. One AS case was detected in an Irish flock with a CS index case however this case was identified via clinical surveillance testing (histopathology and immunohistochemistry) rather than group test analyses and was excluded for this reason from data analyses.

The prevalences of additional AS cases in AS positive flocks were significantly lower than the prevalences of additional CS cases in CS positive flocks (results of the GLMM: OR = 0.03, CI_95%_: 0.02 - 0.05).

### Comparison of prevalence between the surveillance streams

**Figure 1:**
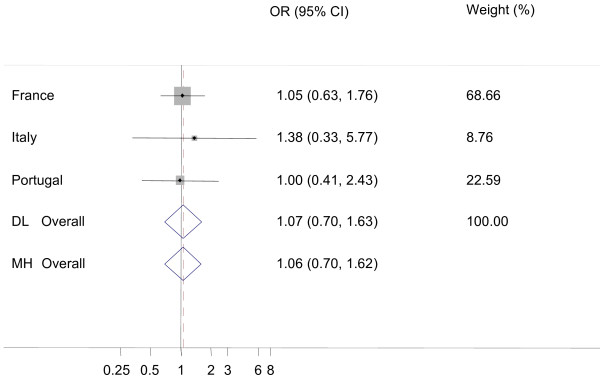
**Forest plot for the probability of additional atypical scrapie (AS) cases in AS positive flocks versus active surveillance in healthy slaughtered animals**. DL: DerSimonian and Laird random effects model, MH: Mantel-Haenszel fixed effects model, Weights are from DL analysis, black dot: country OR, horizontal lines: 95% CI for country OR, grey boxes: country weight, diamond: overall OR with 95% CI, dashed vertical red line: DL overall OR, vertical black line: reference value (1).

**Figure 2:**
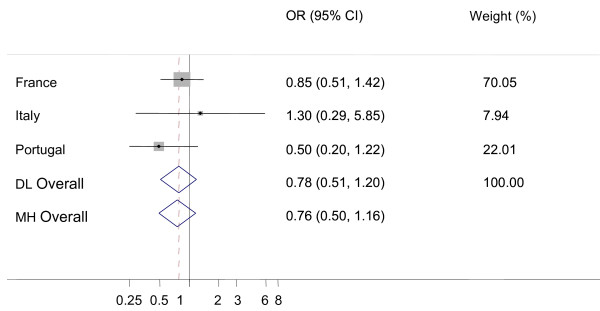
**Forest plot for the probability of additional atypical scrapie (AS) cases in AS positive flocks versus active surveillance in fallen stock animals**. DL: DerSimonian and Laird random effects model, MH: Mantel-Haenszel fixed effects model, Weights are from DL analysis, black dot: country OR, horizontal lines: 95% CI for country OR, grey boxes: country weight, diamond: overall OR with 95% CI, dashed vertical red line: DL overall OR, vertical black line: reference value (1).

Because of small number of additional AS cases, only data from four countries (France, Greece, Italy and Portugal) actually contributed to the meta-analysis and French data contributed the most. None of the comparisons showed any significant heterogeneity among the countries (I-square statistic was never significantly different from zero). The prevalences of additional AS cases in AS positive flocks were not significantly different from prevalences of AS in healthy slaughter (figure 1, Odds ratio (Dersimonian and Laird random effects method, OR_DL _= 1.07, 95% confidence limits (CI_95%_): 0.70-1.63) or in fallen stock (figure 2, OR_DL _= 0.78, CI_95%_: 0.51-1.20).

**Figure 3:**
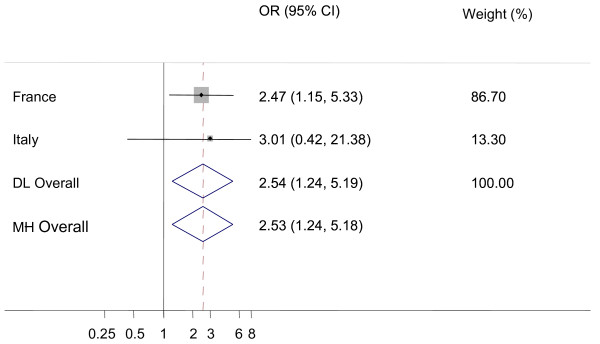
**Forest plot for the probability of additional atypical scrapie (AS) cases in AS positive flocks versus additional AS cases in classical scrapie (CS) positive flocks**. DL: DerSimonian and Laird random effects model, MH: Mantel-Haenszel fixed effects model, Weights are from DL analysis, black dot: country OR, horizontal lines: 95% CI for country OR, grey boxes: country weight, diamond: overall OR with 95% CI, dashed vertical red line: DL overall OR, vertical black line: reference value (1).

**Figure 4:**
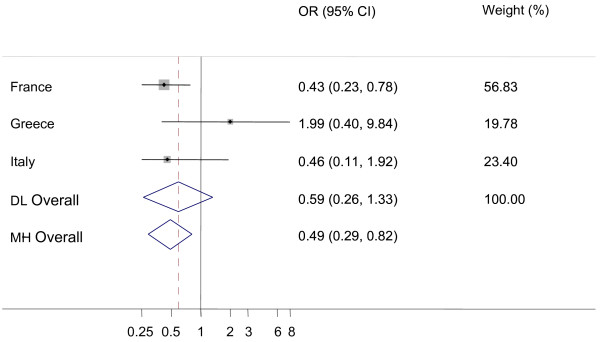
**Forest plot for the probability of additional atypical scrapie (AS) cases in classical scrapie (CS) positive flocks versus active surveillance in healthy slaughtered animals**. DL: DerSimonian and Laird random effects model, MH: Mantel-Haenszel fixed effects model, Weights are from DL analysis, black dot: country OR, horizontal lines: 95% CI for country OR, grey boxes: country weight, diamond: overall OR with 95% CI, dashed vertical red line: DL overall OR, vertical black line: reference value (1).

**Figure 5:**
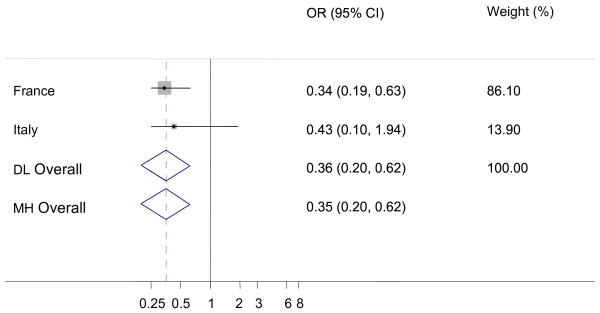
**Forest plot for the probability of additional atypical scrapie (AS) cases in classical scrapie (CS) positive flocks versus active surveillance in fallen stock animals**. DL: DerSimonian and Laird random effects model, MH: Mantel-Haenszel fixed effects model, Weights are from DL analysis, black dot: country OR, horizontal lines: 95% CI for country OR, grey boxes: country weight, diamond: overall OR with 95% CI, dashed vertical red line: DL overall OR, vertical black line: reference value (1).

There was a significantly higher probability of detecting additional AS cases in AS positive flocks compared to CS positive flocks (figure 3) in Italy and in France (OR_DL _= 2.54, CI_95%_: 1.24-5.19). The prevalence of AS among sheep tested due to control measures in CS positive flocks was lower than the AS prevalence in active surveillance (figures 4 and 5). This result was statistically significant for the Mantel-Haenszel (MH) fixed effects models and for the Dersimonian and Laird random effects models applied to fallen stock (figure 4). The funnel plots (not shown) did not reveal associations between results and the size of national sheep populations screened, but few countries contributed to the construction of the plots.

## Discussion

### AS is probably not contagious or much less contagious than CS

Through a large multicentric study, we found that the prevalence of AS among sheep tested during the implementation of control measures in positive flocks was not significantly different from the prevalence of AS in the general sheep population tested through active surveillance. These results suggest that AS does not cluster in positive flocks, and consequently that AS is not contagious or far less contagious than CS. The low odds ratio when comparing the prevalence of additional AS cases in AS positive flocks with the prevalence of additional CS cases in CS positive flocks illustrates the lower frequency of AS aggregation in positive flocks compared with CS. The hypothesis that AS is not contagious has also been supported by reports focussing on case descriptions [11,13,16,19]. Other epidemiological studies also concluded that AS is probably not contagious given the risk factors detected [10,14,15] and one study considering the prevalence of scrapie within flocks [20]. Furthermore, histopathological features suggest that AS may originate spontaneously [2,4,21]. A spontaneous, non-contagious aetiology would not exclude a role for genetics [4] and/or environmental factors in the occurrence of AS [10,14]. The identification of greater numbers of AS cases in flocks wherein animals are exposed to the same factors or where animals have similar genetic background would further evaluate these contributions.

In Ireland one additional AS case was found among a limited number of examined animals, however this single research flock was investigated with an alternative control strategy and one additional AS case was also found in an AS positive flock prior to the period covered by data collected [13]. This data, which was difficult to combine with other countries data, suggests that prevalence of AS in these flocks would be high in comparison with that from the general population. These instances were unique and may have occurred due to chance or to adverse conditions prevailing in these flocks such as high frequency of susceptible animals and/or age structure of the flocks. The fact that additional Irish cases were detected after careful, thorough examination of the cerebellum by histopathology and immunohistochemistry as distinct from routine rapid screening testing[13], stressed the possible underestimation of AS when the cerebellum is not analyzed. As long as the same diagnostic procedures are used in the different streams, the odds ratio of the prevalence in the different streams should not be affected by an underestimation of the prevalence. For the other countries, similar screening procedures were used and so comparisons could be made. As already mentioned there were no indications of a higher prevalence of AS in animals from positive flocks. To improve knowledge of AS it is important that the test procedures are as sensitive as possible to ensure the detection of cases. In this regard, some improvements could be achieved through sampling and testing cerebellum as recommended by EFSA [5,6]. Notwithstanding enhancement of the methods of analysis, the collection of data relative to the flock and the animals tested should also be improved to allow proper interpretation of the results.

Some countries did detect CS cases in AS positive flocks, but not at a higher prevalence when compared with the prevalence of CS detected in active surveillance. Thus there was no indication of a link between the two diseases as supported by a study in Great Britain [20].

The prevalence of AS detected in AS positive flocks was higher than the AS prevalence detected in CS positive in France and in Italy. This observation could result from difference in the genetic structure of the different flocks and/or difference in the age of animals tested, which would need further investigation. Furthermore some of the scrapie cases detected in CS positive flocks were not typed and could actually have been AS cases.

### There was no significant difference of secondary cases prevalence among the different AS control strategies

We did not detect any significant difference in prevalence of additional AS in positive flocks related to the type of control measure applied. However the statistical comparison was only based on data from two countries and thus had low power. Despite absence of significant differences, the control strategies might theoretically lead to different detected prevalences and these should be highlighted. The most important differences between control strategies were the selection of animals for culling and testing, on the basis of genotypes. For classical scrapie all countries had chosen the same genotypes to cull, (based on the genotypes known to be at high risk for classical scrapie). All countries applying selective culling in AS positive flocks (except for Italy and Portugal) culled and tested the same genotypes as in CS positive flocks, even though these do not include all the genotypes at risk for AS (table 2) and on the contrary include genotypes where AS is seldom found. Genetic selection may therefore have affected detected prevalences; a selection of genotypes at risk for CS in both AS and CS positive flocks might have overestimated the sample population prevalence of CS and underestimated this sample population prevalence of AS. In addition there might be a different age structure of the animals tested in different control strategies. When the whole flock or parts of the flock is culled at one point in time, the culled animals which are tested are probably younger compared with animals leaving the flock at the end of their productive lives and tested as part of intensified monitoring. Since AS often is found in older animals, this might theoretically result in detected prevalences being higher for intensively monitored subpopulations. Furthermore, there were different age limits for minimum age of animals tested (12 months or 18 months); if the sample contains a large proportion of young animals this might lower estimated of prevalences. Furthermore some countries reported that it was possible that there was potential misclassification of some of the samples in the data provided (e.g. assigned wrong type of control strategy). However, we have no indication suggesting a differential misclassification leading to bias. Data on the demographic structure (age and genotype) of each flock would have contributed to quantifying the possible effect of these factors, however this data was not available. Theoretically, we would consider that prevalences assessed during intensive monitoring in positive flocks be most comparable to active surveillance prevalences.

### Study at the European level

In this study data from several countries was included and this was facilitated by common EU legislation. However the legislation requires the collection of data on cases only and not on tested animals [18] and therefore data obtained directly from different countries was heterogeneous in presentation and quality resulting from differing national data collation and organisation. Specific assumptions about the similarity of data from different streams were necessary in order to compare prevalences, as has been discussed. In particular it is worth noting that first, despite the lack of significant differences, the control measures were not completely equivalent. Second, the prevalences of AS within healthy slaughter and fallen stock streams between countries were similar but not exactly the same. The differences probably relate to heterogeneity between national sheep populations and/or different strategies for national active surveillance programme implementation as discussed in [12] and [22]. To take these different sources of heterogeneity into account, we considered the use of fixed effect (MH) as well as random effect regression (ML). Results for both methods which were similar indicated that heterogeneity was limited. Results from the countries without any detected additional AS cases in the AS positive flocks were included by adding suitable, differing continuity corrections. In the settings examined, the results were similar and the contribution of the countries without any additional AS case was negligible; this allowed for presentation of more readable results, without continuity correction.

Because important data on the animals tested such as age, PrP genotype and the flock of origin were not available, and because of difficulties diagnosing AS [4], the AS prevalence could have been underestimated. Data on the flock of origin could have also been use to consider the geographical clustering of cases but it was not available. The results for CS, based on data collected in the same way, showed a clear clustering of cases in positive herds.

Since July 2007, EU legislation has allowed for easing of control measures in AS positive flocks. These eased controls require that EU member states, as a minimum, intensively monitor positive flocks through testing of fallen stock and animals leaving the flock for slaughter, but with movement restrictions limited to export interdiction [18]. These changes are based on the rationale that AS is less contagious than CS, which is supported by our results. However, it is difficult to exclude the possibility that AS is naturally transmissible at a low level and precautionary measures including the continued monitoring of animals leaving the flock are sound. The results of surveillance should contribute to determining the nature of control measures that should be employed in the future. Progress has been made, but further improvements in data collection and aligning diagnostic procedures throughout the EU would be required to achieve further results than this study and justify for the cost of testing.

## Conclusion

Within Europe, scrapie surveillance has been intensive for several years and EU legislation requires additional testing in positive flocks. In this study we collected data on active surveillance and surveillance in positive flocks and found that the prevalence of additional AS cases in positive flocks did not differ from the prevalence in active surveillance (healthy slaughter and fallen stock). These results indicate that atypical scrapie does not seem to cluster in positive flocks supporting the hypothesis that atypical scrapie, in contrast to classical scrapie, is not a contagious disease.

## Methods

### Data collection

Since 2002, 28 European countries (25 EU member states plus Iceland, Norway and Switzerland) have been involved in scrapie surveillance in accordance with EC regulation 999/2001 [17] for at least one year. Seventeen countries that had reported at least one case of atypical scrapie from 2002 to 2007 were asked to participate in this study: Belgium, Denmark, Finland, France, Germany, Greece, Hungary, Iceland, Ireland, Italy, The Netherlands, Norway, Portugal, Spain, Sweden, Switzerland and the United Kingdom. In June 2008, a questionnaire was sent to both the Chief Veterinary Officer and the contact persons in the EU Network of Excellence Neuroprion or the former Small Ruminant TSE Network for each country. They were asked to organise for the completion and return of the questionnaire. Two reminders were sent in September and the data was finalised in October 2008.

The questionnaire, which is available on request from the first author, covered information for the period 2002 to 2007 on i) the methods and material used for the detection of AS and CS, ii) the control measures applied in positive flocks, iii) the sampling of animals from flocks under TSE control measures and iv) the sampling of animals in the two streams of active surveillance in the EU; healthy slaughter and fallen stock. During discussions within the working group of the EU Network of Excellence Neuroprion it became clear that data including flock sizes, age and genotype of animals tested was not available from most countries, and consequently we did not collect these data. The test results were reported based on up to date EFSA recommendations for the use of rapid screening tests on relevant biological material (table 1) [5,6]. For each stream and year of the active surveillance programme the numbers of animals tested and the numbers of AS, CS and undetermined TSE index cases were collected. For each control measure and each category of scrapie index case (AS or CS), the number of animals tested and the number of AS, CS and undetermined TSE additional cases were collected. The level of details required by our questionnaire was greater than for animal TSE surveillance EU reports, so participants had to use original source of data (National Reference Laboratory) to answer our questionnaire. We did not examine whether any discrepancies existed between EU reports and our data as a result of differences in data collection.

The strategic control measure options provided in the questionnaire were defined as follows: i) stamping out including all strategies where all animals from the positive flocks were destroyed and a sample of the carcasses of the adults tested, ii) selective culling including all strategies where animals from the positive flocks were genotyped and only animals with certain prion protein genotypes were destroyed and a sample of the carcasses of the adults tested, and iii) intensified monitoring which included the strategies where adults leaving the positive flocks as healthy slaughter or fallen stock were systematically tested but were not culled for the purpose scrapie control/eradication (table 2).

### Data management

The questionnaires were issued in electronic PDF form format (Adobe 8.0 Copyright 1993-2008 Lextek International). Active surveillance data, available from a previous study on prevalence of scrapie [12], was included but could be updated. Data returned was collected in a MS Access database (Microsoft^® ^Access 2003 SP2 Microsoft corporation, WA, USA), and R 2.6 [23] or Stata (Stata Corp. 2007. Statistical Software: Release 10.0. College Station, TX: Stata Corporation) was used for statistical analyses, mapping and graphics. For all statistical tests performed, the significance level was set to 5%.

When the countries returned questionnaires that were partially answered, they were asked to complete their response.

Different AS or CS positive flock national control strategies were grouped in three main categories (table 2). Control strategies that did not suit these categories were reported separately.

The start date for national active surveillance of AS was the first date on which AS could be detected and confirmed or the date from when all re-tested potential AS samples were collected. For statistical analyses, the prevalence of AS was calculated based on data referring to group 1 rapid screening test analyses.

In France additional cases were reported as undetermined TSEs in some CS positive flocks. However, following an examination of a random sample of flocks the authors estimated that AS cases would represent less than 2% of cases and decided to classify undetermined TSE cases as CS for the purpose of analyses.

### Statistical analysis

#### Prevalences

The prevalences defined as the proportions of cases per number of tests, from group 1 for AS and all groups for CS, were estimated with 95% confidence intervals (CI_95%_) using exact binomial method and expressed per ten thousand tests. The prevalences were estimated separately for AS and CS per country and surveillance stream (active surveillance streams and control strategies).

The overall prevalences of AS in active surveillance and the prevalences of AS additional cases in AS positive flocks or in CS positive flocks and the prevalences of CS additional cases in AS positive flocks or in CS positive flocks were estimated by generalised linear regression mixed model (GLMM). More precisely, the total number of expected cases issued from the GLMM was divided by the total number of tests as appropriate for AS or CS.

We tested whether prevalences of AS differed between healthy slaughter and fallen stock streams by using GLMM, using the healthy slaughter stream as reference and accounting for heterogeneity between countries as a random effect.

We assessed whether additional AS case prevalences detected by the differing national control strategies in AS positive flocks were significantly different using a GLMM, accounting for heterogeneity between countries as a random effect and using intensively monitored populations as the reference stream. We conducted the same analyses for additional AS cases detected in CS positive flocks and for additional CS cases detected in CS positive flocks. Since national prevalences of AS in positive flocks were not significantly different we merged them in the rest of the analyses.

Ireland was excluded from the estimation of prevalences of additional AS cases because AS cases were diagnosed using different diagnostic procedures than the other countries: thorough confirmatory diagnostic procedures instead of rapid screening testing. Ireland was also excluded from the estimation of the overall prevalence of additional CS cases because for the earliest datasets a precise figure for the number of animals tested from CS positive flocks was unavailable, however the national prevalences were presented.

Eventually we compared the risk of occurrence of additional cases of scrapie of the same type as the index case, with merging of data from all control strategies, using a GLMM and with countries as random effect and CS as reference.

#### Meta-analysis

We used a meta-analytic approach [24] to summarise and estimate the probability of disease measured by OR. We compared the probability of AS occurring in positive flocks (AS or CS), merging data from different control strategies, with the probability of AS occurring in each stream of active surveillance. We also compared the probability of finding AS additional cases in AS positive flocks with the probability of finding CS additional cases in CS positive flocks, merging data from the different types of control measures. First we used a MH method, assuming homogeneity between countries and we checked this homogeneity by I^2 ^statistic, *i.e*. the percentage of variation attributable to heterogeneity, derived using Cochran's Q [24,25]. Secondly, we fitted random-effects models even when the homogeneity test did not detect significant heterogeneity among countries: we used DL method taking into account the country as a random effect [26]. This last method had the advantage that it weights the relative contribution of each country. We summarized the results in forest plots. We also checked the presence of biases dependant on the number of animals tested using the Egger's regression test^1 ^and funnel plots [25,27].

We considered the use of continuity correction to include countries that had not detected any additional case. In accord with recommendations from literature, we examined options, the first one consisted of systematically added a small constant (k = 0.01) to all counts, the other consisted of adding an empirical continuity correction based on the ratio between the size of the two groups to be compared and the crude ORs as proposed in [28]. In both cases, the constants added were small in order to minimise biases during the estimation of the true OR [26]. Since estimates of the overall OR with continuity correction differed by less than 1% from values without continuity correction and because contributions from countries with zero cases were negligible, results using continuity correction were not presented.

AS data was limited to group 1 analyses and all groups of analyses were considered for CS. Ireland was excluded from this comparison because AS cases were diagnosed using different diagnostic procedures than the other countries as mentioned above.

## Authors' contributions

The project was led by PH and coordinated by AF. The design was decided collectively by all the authors; all the authors contributed to the data collection; AF, MN and ST reviewed the control policy programs; AF, FI and CM analyzed the data; DC, AF, PH, CM, MN, GR, BL discussed the results and all the authors contributed to the manuscript. All authors read and approved the final manuscript.
